# Calculation model for the amount of tradable water rights based on water shortage risk evaluation

**DOI:** 10.1371/journal.pone.0254428

**Published:** 2021-08-25

**Authors:** XiaoYuan Wu, Feng-Ping Wu, Xia Xu, Fang Li

**Affiliations:** 1 Business School, Hohai University, Nanjing, China; 2 National Engineering Research Center of Water Resources Efficient Utilization and Engineering Safety, Nanjing, China; University of Defence in Belgrade, SERBIA

## Abstract

A scientific measurement of the amount of tradable water rights forms the premise for reaching an agreement in water rights trading. However, the existing measurement methods, based on water saving potential, still have problems caused by large computation workload and difficult-to-control errors. Conflicts also easily emerge between transferor and transferee during the execution of transaction agreements. This paper proposes a new method for measuring tradable water rights from the perspective of an assessment of the risk of water shortage for the transferor. The following describes the basic idea: An index system is established for the assessment of the water shortage risk of the transferor to identify water shortage risk categories. The impact of the water rights transaction volume on the transferor’s water shortage risk category is analyzed under different incoming water frequencies. The transferor’s water shortage risk threshold is set and a simulated annealing algorithm is designed to calculate the theoretical value of tradable water rights. The following summarizes the innovation of the proposed method: The water resource shortage risk evaluation index of the transferor is constructed based on water resource endowment, water supply, water demand, and water ecological environment of the transferor; then, a risk classification evaluation model of water resource shortage is established and a measurement method of tradable water rights is introduced. Comprehensive analysis of a case analysis of Helan County in the Ningxia Autonomous Region, China, shows that the recommended value of tradable water rights of Helan County is 40 million m^3^. Various methods are used to calculate the weights of evaluation indexes, which are compared to the measured results of tradable water rights; moreover, the sensitivity of the results is analyzed. The obtained results show that the use of water-saving potential to measure the amount of tradable water rights is feasible.

## 1. Introduction

### 1.1 Research background

In to optimize the redistribution of water resources and improve the efficiency of water use, the Chinese government actively encourages the development of water rights trading through market mechanisms. In 2012, the Chinese government proposed to actively conduct trials of trading water rights and pollution emission rights. In 2017, the government clearly proposed to "accelerate the construction of water rights markets, promote the confirmation and entry transactions of water resources use rights". Furthermore, the following requirement was stipulated: "the market needs to play a decisive role in resource allocation [1]".

According to national requirements, the Ministry of Water Resources and other relevant ministries actively promote transactions of water rights. In 2014, the Ministry of Water Resources issued the "Notice on Carrying out Water Rights Pilot Work", and initiated pilot work in Ningxia, Jiangxi, and five other provinces. These pilot projects provided experience and references for the national promotion of the construction of an appropriate water rights system. In 2016, the Ministry of Water Resources issued “the Interim Measures for the Administration of Water Rights Transactions”, which clarified the main forms of water rights transactions. These include regional water rights transactions, water withdrawal rights transactions, and water rights transactions of irrigation water users. In 2016, the Chinese water rights exchange was put into operation. In 2018, nine departments, such as the National Development and Reform Commission and the Ministry of Finance, jointly issued the "Action Plan for Establishing a Market-oriented and Diversified Ecological Protection Compensation Mechanism". This action plan proposed to encourage and guide water rights trading as well as to improve existing water rights trading platforms. According to statistical data, since 2016, more than 500 water rights transactions have been processed through the China water rights exchange. This means that Chinese water rights trading has entered a good stage of development.

The scientific calculation of the amount of tradable water rights is one of the core requirements of water rights trading. Together, the amount of tradable water rights, transaction actors, water rights transaction period, and standard water measurement constitute the four basic elements that affect water rights transaction prices [2]. The amount of tradable water rights refers to the amount of water resources, the transfer of which needs to be clarified in the water rights transaction agreement. For the transferor, under the premise of satisfying the transferor’s own production, living, and ecological water use, the amount of tradable water rights represents the maximum amount of water rights that can be sold through the water rights trading market. For the transferee, the amount of tradable water rights is the maximum amount of water rights purchased through the water rights trading market to compensate for the gap in water demand for production, life, and ecology.

The determination of the amount of tradable water rights must consider the natural and social capacities of both parties of the transaction, especially the transferor’s water consumption of the social and economic development and ecological safety. If the amount of tradable water rights to be traded between both parties is too large, it is very likely that a shortage of water resources will affect the social and economic development of the transferor in the future. Moreover, in serious cases, such a situation may cause disputes, and even damaged the durability and stability of the water rights transaction market. Therefore, studying the quantitative method of the scientific measurement of tradable water rights is not only conducive to determining the scale of water rights traded by both parties, but also to the sustainable development of the water rights trading market.

### 1.2 Research question

How to scientifically measure the amount of tradable water rights is a difficult issue that must be overcome for the development of water rights trading. At present, the method of using the transferor’s water saving potential for measuring the amount of tradable water rights has been generally accepted by the academic community [3]. This method offers the advantages of clear logical thinking and good interpretability. However, the application of this method faces two difficulties: the first difficulty is the involvement of many links and parameters in the measurement of the water-saving potential, which restricts the accurate measurement by many objective and subjective factors. Furthermore, the calculation is often too work intensive; the second difficulty is that while the water-saving potential is calculated, the proportion of water-saving potential that can be used for water rights transactions varies between different regions. The purpose of water rights trading is the improvement of the utilization efficiency of water resources. An unreasonable proportion of water-saving potential used for water right transaction can easily cause calculation errors, and may cause conflicts between the transferor and the transferee in the execution of the transaction agreement.

The basic premise of water rights trading is a difference in the marginal benefits of different entities that use water resources. The process of water rights trading is actually a process in which water use rights are transferred from the party with lower marginal revenue to the party with higher marginal revenue.

In general, the amount of tradable water rights agreed upon in the water rights transaction agreement mainly depends on the amount of tradable water rights the transferor can transfer. In the water rights transaction process, the legal rights of the transferee must be protected. Furthermore, the transferor’s water shortage risk cannot be increased. Therefore, measuring the amount of tradable water rights from the perspective of controlling the risk of water shortages the transferor faces is more conducive to safeguarding the rights and interests of both parties of the transaction, and can avoid potential risks caused by the transaction process. The present investigation showed that since China’s water right trading market is not yet complete, disputes between both parties of the execution of the transaction agreement often occur because of problems associated with the transaction volume and the transaction price. A case of water rights trading between DongYang and YiWu, Yiwu City (the transferee) yielded the permanent use rights of 50 million m^3^ of water resources per year from DongYang City (the transferor) for a one-time payment of 200 million CNY. In recent years, a number of people in DongYang, i.e., the transferor, have begun to believe that water rights trading has damaged their long-term interests, which has caused conflicts between both sides and led to the emergence of hidden dangers of social instability. Therefore, studying the measurement method of tradable water rights from the perspective of avoiding the water risk of the transferor has both theoretical and practical significance.

### 1.3 Literature review

How to determine the amount of tradable water rights is one of the key technical issues of the water rights trading mechanism. In recent years, with the development of a number of water rights trading practices, scholars have begun to conduct research on the tradable water rights from multiple angles: (1) Foreign scholars mainly studied the analysis methods of the amount of tradable water rights based on restrictive factors. Marino and Kemper [4] suggested that the establishment of water rights transaction must have sufficient information resources of the amount of tradable water rights and necessary infrastructure for measuring and transferring the traded water resources. Matthews et al. [5] showed that water rights trading for the transfer of agricultural irrigation water to industrial water will change river runoff and aggravate droughts during dry periods. Dellapenna [6] suggested that water rights trading may impact the ecological environment, economic development, and the lives of residents at water rights transfer sites. Therefore, Georgia stated that the amount of tradable water for cross-basin water rights trading must be the remaining water volume after meeting the water demand of the river basin. Guo et al. [7] jointly analyzed water rights trading and water conservation management and found that they are highly compatible and related. Moreover, it was also found that the combination of water rights trading and water conservation management can promote the implementation of water conservation management contracts. To accurately quantify the comprehensive value of water resources, based on market and administration, Danyang Di et al [8] proposed a water rights transaction game for optimizing the water transaction volume and transaction price for each region of the Yellow River Basin. Shen [9] proposed the concept of standard water, established a standard water quantity measurement model, converted the exchanged water quantity into a standard water quantity, and used DongYang and Yiwu as case studies to calculate the water rights transaction amount. The results help to ensure the fairness and permanence of the water rights transaction process. (2) Chinese scholars have also conducted theoretical and quantitative research on the amount of tradable water. Wu [10] established a two-stage water rights transaction equilibrium price calculation model based on shadow price theory. In their model, the first stage is the unilateral measurement stage, which constructs a shadow price model from both the transferor and the transferee. The second stage is the bilateral coupling measurement stage, which aims to maximize the overall benefit of the national economy, and uses the differential game equilibrium theory to determine the equilibrium water price. Hu et al. [11] proposed to use actual water users as subjects of water rights, and to change the water withdrawal system to a water consumption right system; moreover, they clearly defined water rights as water consumption rights, thus clarifying the ownership of property rights. Li et al. [12] considered that the amount of water rights allowed to be traded and how to calculate the volume of water taken in actual transactions is one of the key technical issues of the water rights trading mechanism. Under the premise of a given water use efficiency, by analyzing the industry’s advanced water-saving technologies (both in China and internationally), comparing existing and planned water-saving indicators, and analyzing the water-saving potential of specific regions and industries, the theoretical tradable water volume can be obtained. Zhao et al. [13] used the maximum overall economic and social benefits of a river basin as objective function, and applied the control of total water consumption and water loss as constraints to construct a water withdrawal right transaction model. By taking the Shaying River Basin as example, this model was used to optimize the second water rights transaction scheme. Tan [14] considered the difference in water guarantee rates between agriculture and industry, as well as the encroachment of industrial water on agricultural water; then, the amount of tradable water was calculated and converted from agricultural water to industrial water. Liu et al. [15] constructed a water rights transaction decision-making model, selected water rights transaction behaviors, made transaction decisions, and analyzed the potential water rights transaction volume in the study area. Their research assumed that decision-making behavior is constrained by the maximization of economic benefits of the main water body, and is also affected by the cost of water saving and the benefits obtained following the transaction. The research results provide a basis for the construction of the regional water rights market and the determination of the tradable water volume in the regional water market. Su [16] summarized the implementation of water rights conversion pilot projects in Ningxia and the Inner Mongolia Autonomous Region of the Yellow River Basin, and evaluated it from the four aspects of society, economy, ecology, and theory. Finally, the characteristics, problems, and prospects of the Yellow River water rights conversion scheme ware identified.

The above analysis showed that existing research on the amount of tradable water rights can be divided into two categories: ① With regard to qualitative research, existing research mainly focused on the control of the trading market, as well as the design of the trading mechanism and ownership management. ② With regard to quantitative research, foreign scholars studied methods for measuring the amount of tradable water rights mainly from the remaining water after meeting the water demand of the river basin; domestic scholars mainly studied this amount from the water saving potential.

Research on how to measure the amount of tradable water rights based on the change of water shortage risk has not yet been reported. Changes in the water supply and water demand of an area may change the water shortage risks of this area, which would then affect the socio-economic and ecological security of that area. This means that water rights transactions will inevitably cause changes in the transferor’s water supply to ensure that own needs are met. Consequently, the amount of tradable water rights should be adjusted according to the water shortage risk of the transferor.

### 1.4 Research idea

This paper proposes a new method for estimating the amount of tradable water rights. The basic principle is to measure the amount of tradable water rights under the premise of controlling the risk that the transferor experiences water shortage. In theory, this approach compensates for the deficiency of existing calculation methods such as large amounts of required calculations and calculation errors. In practice, this approach is more conducive to safeguard the rights and interests of both parties and to avoid potential risks associated with the water supply in the transaction process.

The steps of this model are summarized in the following:

Step 1: The risk evaluation index of water shortage of the transferor is constructed. Here, the combined method is used to measure the weights of different indicators. A fuzzy comprehensive evaluation model is used to evaluate the risk categories of water resources shortage.
Step 2: The mid-term and long-term water shortage risk thresholds of the transferor are set. According to the water inflow frequencies of 50%, 75%, and 90%, the evaluation index value of the planning year in class A and class B is predicted by different methods. The simulated annealing algorithm is used to calculate the theoretical value of tradable water rights.
Step 3: Combined with the case study of Helan County in Ningxia Autonomous Region, China, the recommended value of tradable water rights of this County is obtained by comprehensive analysis.
Step 4: Discussion and analysis of the results. To verify the robustness of the model, various methods were used to calculate the weights of evaluation indexes and to compare the calculation results of tradable water rights. To verify the feasibility of the proposed method, the results are compared with the obtained conclusions when the water-saving potential is used.

**Fig 1:**
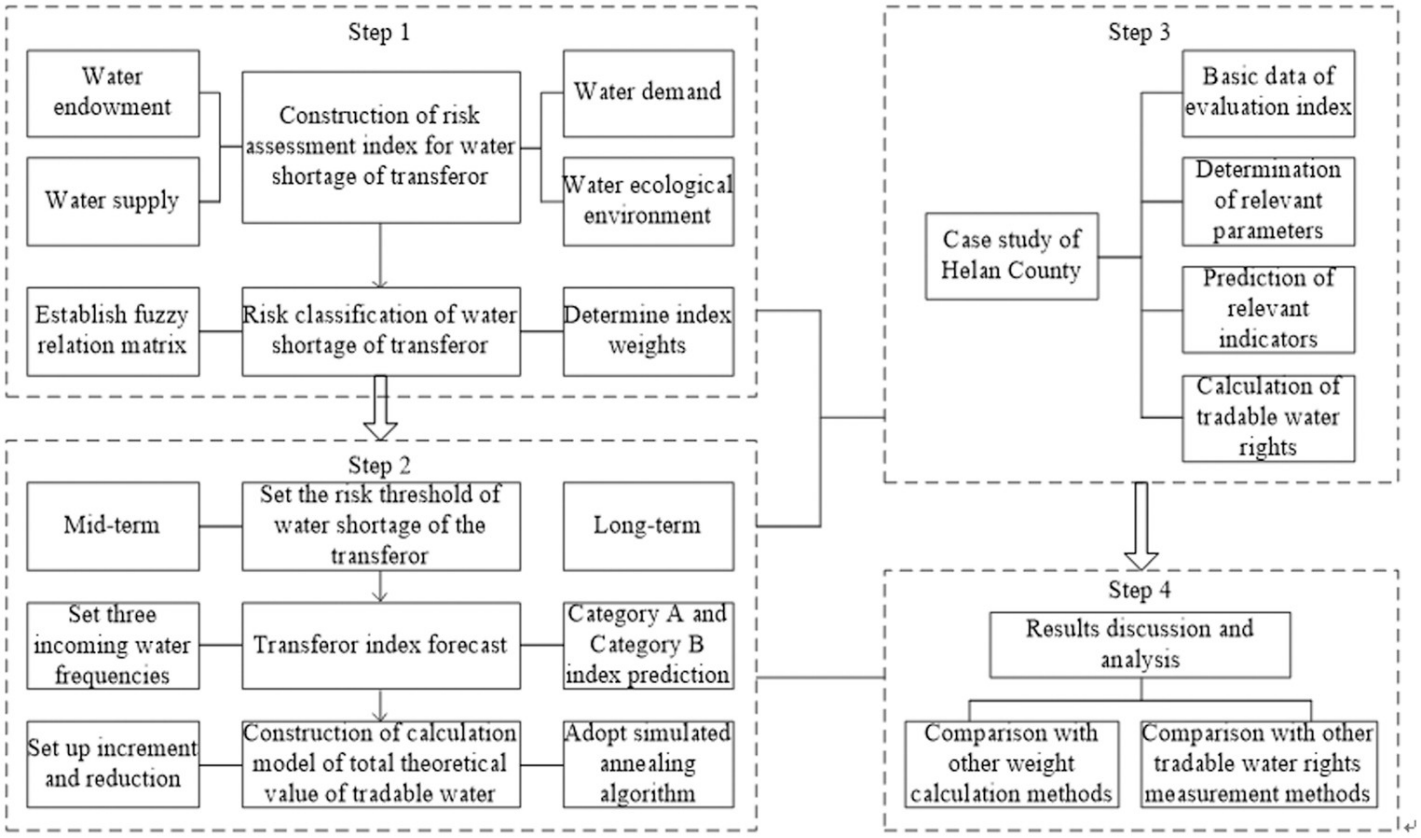
Basic flow chart of the proposed method.

A basic flow chart of the proposed method is shown in Fig 1.

The innovation of this work is reflected in to following: A new method for measuring tradable water rights is proposed from the perspective of assessing the water shortage risk of the transferor. Specifically, the water shortage risk evaluation index of the transferor is constructed from the following four factors: natural and social conditions of water resources of the transferor, water supply capacity, water demand degree, and regional water ecological environment change trend. The fuzzy comprehensive evaluation method is used to construct the risk classification evaluation model of water resource shortage. A tradable water rights measurement method is proposed based on a simulated annealing algorithm.

The remainder of this paper is organized as follows: Section 2 introduces the study area. Section 3 introduces the methods. Section 4 analyzes the results. Section 5 discusses the findings and Section 6 concludes the study.

## 2. Case study

Helan County of Ningxia Autonomous Region, China, is located in the central and northern parts of the Qingtongxia irrigated area. Helan County is located 8 km from Yinchuan urban area, with a land area of 1,595.5 km^2^. Its geographical coordinates range between 105°53’-106°36’ east longitude and 38°26’-38°48’ north latitude. The altitude of Helan Mountain in the west exceeds 1400 m, the piedmont floodplain at the eastern foot of Helan Mountain is 1122–1400 m above sea level, and the modern alluvial plain of the Yellow River in the East is 1102–1122 m above sea level.

In recent years, Helan has exerted great efforts to build a demonstration county for the comprehensive reform of modern ecological irrigation area and agricultural water price. Moreover, Helan achieved remarkable results in water-saving renovation and construction. At present, Helan County is transferring part of the use right of the Yellow River water through water rights trading, thus further broadening its financing channels for the construction of modern ecological irrigation areas. Therefore, Helan County was used as a case study.

The details of the water rights trading in Helan are as follows: transferor: Helan People’s government; transferee: enterprises that need to solve the industrial water consumption through water rights trading in Ningxia; trading volume: 10^7^ m^3^; trading period: 25 years; trading price: 1.094 CNY/m^3^; payment mode: enterprises can choose to pay water rights trading costs annually or as a one-time payment according to their own operational conditions [17]. Since the water rights trading period of Ningxia is basically set for 25 years, the water rights trading in Helan will be implemented from 2020; thus, the trading period was set as 2021–2045.

## 3. Methods

### 3.1. Evaluation model of transferor’s water shortage risk

#### 3.1.1. Establishing the transferor’s water shortage risk evaluation index system

According to Malthus’s Absolute Resource Scarcity Theory and Ricardo’s Relative Resource Scarcity Theory [18], the scarcity of water resources can be classified into absolute scarcity and relative scarcity. The natural endowment of water resources is the main source of its “absolute” scarcity. Because of the rapid socio-economic development over recent years, water resources are increasingly "relatively" scarce. The two main reasons for their relative scarcity are: first, the imbalance between supply and demand, and second, with continuously increasing utilization of water resources, the discharge of sewage also increases, which affects the quantity of available high-quality water resources and causes the scarcity of water resources.

Based on the above analysis, and related literature [19–24] about the water shortage risk evaluation index system, the evaluation index system of transferor’s water shortage risk was divided into the following four aspects: natural and social conditions of water resources, supply of water resources, demand for water resources, and the ecological water environment, as shown in Table 1.

**Table 1:** Index system of transferor’s water shortage risk.

Factor	Detailed indicator	Label/unit	Measurement method/data source	Indicator type
Natural and social conditions of water resources	Per capita water resources	*C*_1_ (m^3^/person)	Total water resources / total population	–
	Water production modulus	*C*_2_ (m^3^/km^2^)	Total water resources /total land area	–
	Aridity index	*C*_3_ (-)	Evaporation /simultaneous precipitation	+
	Urbanization rate	*C*_4_ (%)	Urban population /total population	+
	Population density	*C*_5_ (person/ km^2^)	Total population / total land area	+
Supply of water resources	Water supply per capita	*C*_6_ (m^3^/person)	Available water supply / total population	–
	Water supply rate	*C*_7_ (%)	Water supply / total water resources	–
	Surface water supply ratio	*C*_8_ (%)	Total surface water / total water supply	–
	Ground water supply ratio	*C*_9_ (%)	Total ground water / total water supply	–
Demand for water resources	Per capita water consumption	*C*_10_ (m^3^/person)	Total water consumption / total population	+
	Effective utilization coefficient of farmland irrigation water	*C*_11_ (-)	Net water consumption of crops / total water diversion from canal head	–
	Water consumption of 10^4^ CNY	*C*_12_ (m^3^/10^4^CNY)	Industrial water consumption / industrial added value	+
	Water consumption rate	*C*_13_ (%)	Water consumption / total water consumption	+
Ecological water environment	Water function area compliance rate	*C*_14_ (%)	From “Water Resources Bulletin”	–
	Industrial pollution ratio	*C*_15_ (%)	Industrial wastewater discharge/total water supply	+
	Life pollution ratio	*C*_16_ (%)	Domestic wastewater discharge/total water supply	+

Note: The index type "+" implies that the greater the index value, the greater the degree of water resource scarcity, while the index type "–" means the opposite.

Of the factors listed in Table 1, after the transaction of water rights, the transferor’s "water resources natural and social conditions" and "water ecological environment" factors will generally not change because of the amount of traded water rights. The indicators "water supply per capita" and "water supply rate" under the "water resources supply" category will be directly and negatively affected by the trading of water rights. The "surface water supply ratio" and "ground water supply ratio" will not show obvious changes. Furthermore, the water rights transaction is bound to encourage the transferor to conduct positive water-saving policies, and to restrain water resource demand indicators. Based on the above analysis, this paper focuses on the impact of tradable water rights on transferor’s "water supply per capita" and "water supply rate" indicators. For the prediction of relevant indicators the influence of tradable water rights on water resource demand will be appropriately adjusted by parameters.

#### 3.1.2. Classification of the water shortage risk of the transferor

To directly reflect the degree of the water shortage risk of the transferor, it is necessary to evaluate the level of water shortage risk. Therefore, the fuzzy comprehensive evaluation model was used to analyze the water shortage risk. The following steps were implemented:

1. The evaluation step for the water shortage risk’s classification of the transferor
  ① Establishing the factors domain of the transferor water shortage risk: *U* = {*c*_1_, *c*_2_, ⋯, *c*_*n*_}
  ② Establishing the domain of water shortage risk assessment level: *V* = {*v*_1_, *v*_2_, ⋯ *v*_*m*_}
  ③ Establishing the fuzzy relation matrix between *U* and *V*:
    $$R=\left[\begin{array}{cccc} r_{\text{11}} & r_{\text{12}} & \cdots & r_{1m}\\ r_{\text{21}} & r_{\text{22}} & \cdots & r_{2m}\\ \vdots & \vdots & \vdots & \vdots \\ r_{n1} & r_{n2} & \cdots & r_{nm} \end{array}\right]$$ (1)
    Where, *r*_*ij*_ represents the relative membership of the factor *i* in *U* to the j grade of *V*_j_.
  ④ Determining the risk level of water shortage. The fuzzy comprehensive evaluation model for the risk assessment of water resources shortage is the synthetic operation of *W* and *R*, namely:
    B=W∘R (2)
    Where, *W* = (*w*_1_, *w*_2_, ⋯, *w*_*n*_) represents the weight of water shortage risk index, and satisfies $\sum_{i=1}^{n}w_{i}=1$. The index weight was determined by the combination method. “∘” is the fuzzy composition operator, and the weighted average operator M(∘,⊕) was used for the calculation. B is the evaluation result set of the water shortage risk $b_{j}=\sum_{i=1}^{n}w_{i}r_{ij}\left(j=1,2,\cdots ,m\right)$, and the corresponding results of max *b*_*j*_ are selected as the final evaluation grade *v*_*j*_.
2. Determination of the relative membership degree
  The evaluation grade is divided into five grades, according to their value, as lower, low, medium, high, and higher values. The value of index *c*_*i*_ is *x*_*i*_, and the five standard values of the evaluation domain *V* are *S*_*i*1_, *S*_*i*2_, …, *S*_*i*5_. The linear membership function was used to determine the membership of each index.
  ① When *x*_*i*_ ≻ *S*_*i*1_, where "≻" means "better than", and corresponding to the indicator with indicator type of "-", this means “>”, otherwise, it means “<”. Order
    $$r_{i1}=1;r_{ij}=0,j=2,3,4,5$$ (3)
  ② When *x*_*i*_ ≺ *S*_*i*5_, where "≺" means "inferior to", and corresponding to the indicator with indicator type of "-", this means “<”, otherwise, it means “>”. Order
    $$r_{ij}=0,j=1,2,3,4;\;r_{i5}=1$$ (4)
  ③ When *S*_*i*,*k*+1_ ≺ *x*_*i*_ ≺ *S*_*ik*_, *k* = 1, 2, 3, 4. Order
    $$r_{ik}=\left|\frac{x_{i}-S_{i,k+1}}{S_{k}-S_{i,k+1}}\right|;r_{i,k+1}=1-r_{ik};r_{ij}=0;j\neq k\;\text{or}\;k+1$$ (5)
3. Determination of indicator weights.
  This paper suggests that the judgment of the importance of the water resource shortage risk assessment index should not only consider the inherent law between the index data, but should also fully consider the experts’ understanding of the importance of the index. Therefore, the combined subjective and objective method was used to calculate the weights of different indicators.
  ① Determination of subjective weight $w_{j}^{\left(1\right)}$.
    A hierarchical structure model was established, and the risk assessment of water shortage was taken as the target layer. The criterion layer was established from four dimensions: natural and social conditions of the water, water resource supply, water resource demand, and water ecological environment. The index layer is composed of (*C*_1_, *C*_2_, …, *C*_16_) corresponding to the criteria layer.
    Ten experts (mainly from the China Water Rights Exchange, local water resources trading center, university professors, and the water administration department) were invited to assign importance degrees to the indicators according to the five-scale method. A pairwise judgment matrix was obtained. Then, the subjective weight $w_{j}^{\left(1\right)}$ was calculated by the root method.
  ② Determination of objective weight $w_{j}^{\left(1\right)}$.
    The variation coefficient of each index was calculated to measure the value difference degree of each index.
    $$e_{j}=\frac{S_{j}}{\bar{x_{j}}}$$ (6)
    Where, *e*_*j*_ represents the variation coefficient of index J, *S*_*j*_ represents the standard deviation of index J, and $\bar{x_{j}}$ represents the average of the index J. The objective weight of each index was calculated as shown in Formula (7):
    $$w_{j}{}^{\left(2\right)}=\frac{\;e_{j}}{\sum_{j=1}^{n}e_{j}}$$ (7)
  ③ Determination of the combined weight
    *W*_*j*_(*j* = 1, 2, …, *n*) was set as combined weight. The specific algorithm is presented in the following:
    $$w_{j}=\frac{w_{j}^{\left(1\right)}w_{j}^{\left(2\right)}}{\sum_{j=1}^{n}w_{j}^{\left(1\right)}w_{j}^{\left(2\right)}}$$ (8)

### 3.2. Method for measuring tradable water rights of the transferor

#### 3.2.1. Setting the water shortage risk threshold of the transferor

The basic principle of determining the quantity of tradable water rights from the transferor is that the risk of water shortage of the transferor should be maintained within a controllable range during the period of water right transaction.

Based on the above analysis, to distinguish the water shortage risk of the transferor in the planning year, the transferor’s water shortage risk threshold was set as following: the water shortage risk level of the transferor is maintained at the "low" level in the planning year (mid-term), and below the "low" level in the planning year (long-term). The corresponding grade of max *b*_*j*_ is *v*_*k*_; therefore, the criteria can be constructed as follows:

$$\left\{\begin{array}{ll} v_{k}=v_{1} & ,mid-term\\ v_{k}\in \left\{v_{1},v_{2}\right\} & ,long-term \end{array}\right.$$ (9)

#### 3.2.2. Determination of the tradable water rights based on the simulated annealing algorithm model

**Fig 2:**
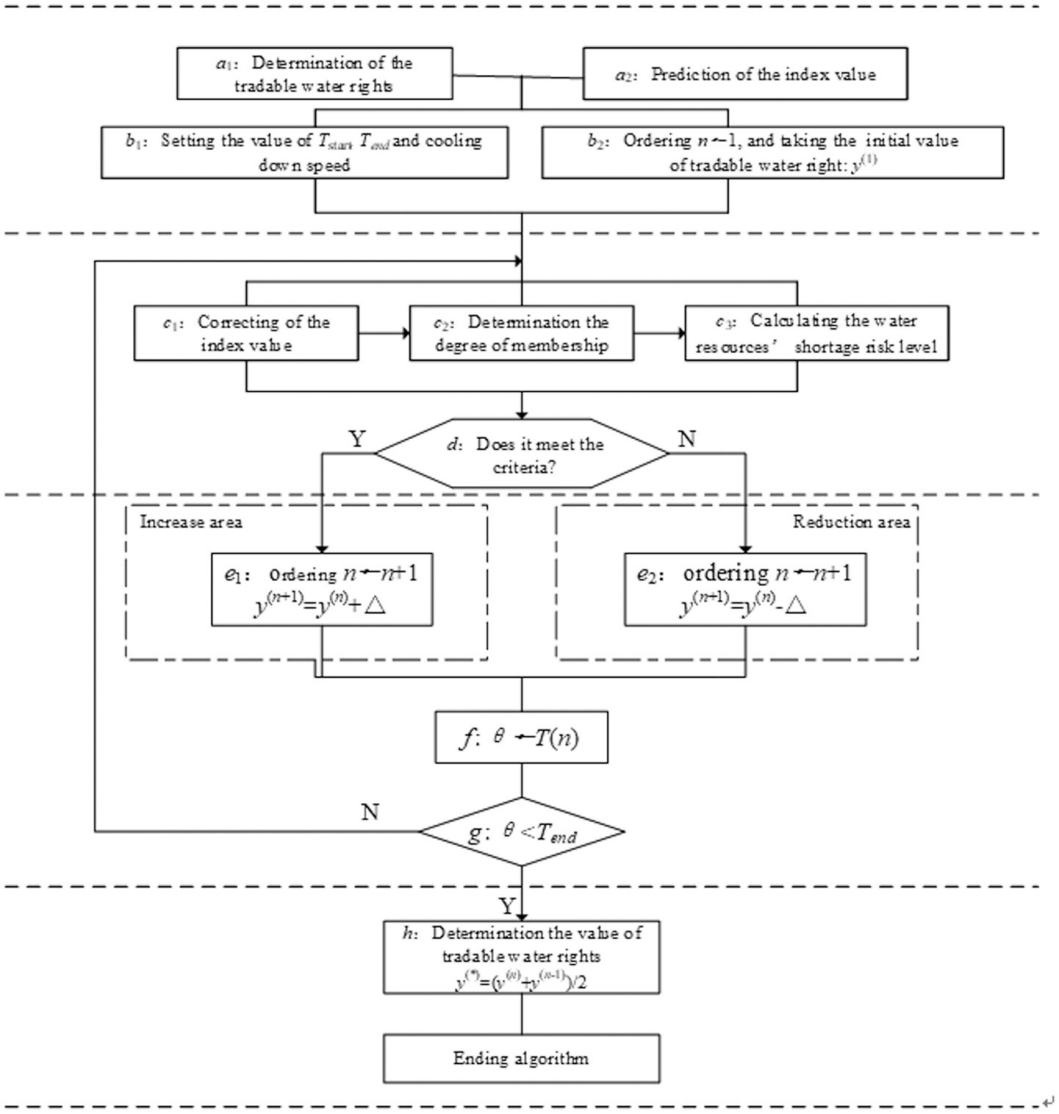
Flow chart of the simulated annealing algorithm for calculating tradable water rights.

The simulated annealing algorithm is a stochastic optimization algorithm based on the Monte Carlo iteration strategy. The random solution generated by the simulated annealing algorithm starts from a higher initial temperature, and finds the global optimal solution in the solution space with the continuous decrease of temperature [25]. This paper designed 16 indexes to measure the water resources shortage risk. Among them, the indexes of "water supply per capita" and "water supply rate" change with changing tradable water rights, which then influences the risk level of water resource shortages. In this paper, the measurement of tradable water rights represents an attempt to investigate the change of the water resources shortage risk level through the change of tradable water rights. It satisfies the mechanism of the simulated annealing algorithm based on metropolis criterion to explore the target. Moreover, in the process of using the simulated annealing algorithm to calculate tradable water rights, this paper designs the situation of "increasing" or "decreasing" the tradable water rights, to avoid the algorithm falling into local optimization. The algorithm includes four core steps, and the specific process is shown in Fig 2.

Step 1: "Start". Data is imported and the model is initialized. Formulas (6)–(8) are used to determine the index weight (*a*_1_ in Fig 2) and predict the index value in the case of no water right trading in a year and a certain frequency of water (*a*_2_ in Fig 2). Setting the parameters of the simulated annealing algorithm (*b*_1_ in Fig 2), where the initial temperature is T_*start*_ and the end temperature is T_*end*_. The cooling equation is as follows:
  $$T\left(n\right)=T_{start}/\text{lg}\left(1+n\right)$$ (10)
  Setting *θ* ← *T*_*start*_, and the initial value of cycle count *n* ← 1. An initial value of tradable water right is randomly generated, and is denoted as *y*^(1)^(*b*_2_ in Fig 2), and is used as current optimal solution.
Step 2: "Judgment", judging whether the new plan can be accepted. For the current tradable water rights *y*^(*n*)^, the affected index values can be calculated (*c*_1_ in Fig 2). Then, the impacts of the change of tradable water rights on the "water supply per capita" and the "water supply rate" are analyzed. The specific formulas are shown in the following:
  $$C_{6}=\left(W_{\text{supply}}-y^{\left(n\right)}\right)/P_{\text{total}}$$ (11)
  $$C_{7}=\left(W_{\text{supply}}-y^{\left(n\right)}\right)/W_{\text{total}}$$ (12)
  Where, *y*^(*n*)^ represents the current (nth round) tradable water rights assignment, *P*_supply_ represents the total population of the region, and *W*_total_ represents the total regional water resources.
  Using Formulas (3)–(5), the membership degree can be determined according to the standard value of the evaluation grade (*c*_2_ in Fig 2). Formula (2) is used to calculate the transferor’s water resources shortage risk level (*c*_3_ in Fig 2), and diagnose it by using the discriminant criteria set in Formula (9): if the condition (9) is met, the new scheme will be "accepted"; if the condition (9) is not met, the new scheme will be "rejected".
Step 3: "Adjustment", this paper adjusts and optimizes the scheme through iteration. According to the diagnosis results, if the new scheme is "accepted", the iterative process will be automatically transferred to the "increase area" of tradable water rights; however, if the new scheme is "rejected", the iterative process will be automatically transferred to the "reduction area" of tradable water rights. The order is *n* ← *n* + 1, and a new scheme can be obtained by "increasing" (e_1_ in Fig 2) or "reducing" (e_2_ in Fig 2) the currently tradable water rights. The adjustment formula of the new scheme is shown as Formula (13):
  $$y^{\left(n\text{+}1\right)}=\left\{\begin{array}{l} y^{\left(n\right)}\text{+}\Delta ,\;increase\;area\\ y^{\left(n\right)}-\Delta ,\;reduction\;area \end{array}\right.$$ (13)
  where, Δ represents the increment or the reduction, and is predetermined according to the allowable deviation of the tradable water rights.
  The order is *θ* ← *T*(*n*) (*f* in Fig 2). Judging the relationship between *θ* and *T*_*end*_, if *θ* ≥ *T*_*end*_, Step 2 and Step 3 are repeated to accept the next round of diagnosis, if *θ* < *T*_*end*_, then go to Step 4 and output the current scheme as optimal scheme.
Step 4: "Output", outputting the scheme by taking the average value of two rounds as the theoretical value of tradable water rights (*h* in Fig 2):
  $$y^{\left(*\right)}=\left(y^{\left(n\right)}\text{+}y^{\left(n-1\right)}\right)/2$$ (14)

#### 3.2.3. Determination of relevant parameters

1. Different water inflow frequencies. To analyze the change of the transferor’s water shortage risk level after having implemented the water right transaction in a planning year, the planning year is recorded as the kth year, and three types of water inflow frequency years are calculated. These are a normal water inflow year (where the inflow frequency is 50%), a biased dry year (where the inflow frequency is 75%), and an extremely dry year (where the inflow frequency is 90%).
2. Planning year. Here, "medium term" is defined as the 10th year after the implementation of water rights transaction, and "forward term" is defined as the 20th to 25th year after the implementation of the water rights transaction, or as the termination year of water rights trading period, as stipulated in the contract.
3. Determination of increment or decrement (Δ). The larger the Δ value, the less iteration cycles are used; however, this it can affect the calculation accuracy of the theoretical value of tradable water rights. In contrast, the smaller Δ, the more iteration rounds, which can improve the accuracy of the calculation. This paper provides two ideas to determine the value of Δ: ① The equivalent method, which means that the value of Δ remains the same in each round, and can be determined according to the accuracy requirements of experts on the theoretical value of tradable water rights. Moreover, it is suggested that Δ should be twice the allowable deviation of tradable water rights. ② The descending method, which means the value of Δ in each round is gradually decreasing. It is suggested that the first adjustment increment or decrement value (Δ_1_) should be 5–6 times of the allowable deviation of tradable water rights; then, the Δ_*n*+1_ value of each round is 0.8 times of that of the previous round, i.e., Δ_*n*+1_ = 0.8Δ_*n*_. In this way, after 4–5 rounds of iterations, the calculation error of tradable water rights can be controlled to meet the requirements of the allowable deviation.
4. Division of evaluation indexes corresponding to the grading interval of evaluation standard value. Evaluation indexes can be divided by referring to relevant national standards, by combining regional water resources and social-economic characteristics, or by referring to existing literature and expert opinions.

#### 3.2.4. Index prediction

To calculate the risk level of water shortage of the transferor in a planning year (i.e., the kth year), it is necessary to predict the evaluation index of the planning year, and to analyze the change of the index value according to the three types of water inflow frequencies. Moreover, to improve the accuracy of the prediction, this paper divides the evaluation indicators into two categories based on data availability. These are indicators A and B. Category A indicators can be directly obtained (or their development trend can be estimated) based on the annual average value, as well as relevant national and local planning reports. For category B indicators, it is generally impossible to directly obtain forecast data through planning reports; therefore, reasonable methods should be adopted for their prediction. Moreover, as the specific prediction method of category B index is related to the case, the specific prediction model is proposed based on the sample data and the characteristics of the case in this paper. The specific division of category A and B indicators is shown in Table 2.

**Table 2:** Prediction methods of different evaluation indexes.

Index category	Index code	Prediction methods
Category A	*C*_1_, *C*_2_, *C*_3_, *C*_8_, *C*_9_	The long sequence samples were analyzed, and the predicted values are mainly determined according to the average value of many years
	*C*_4_, *C*_5_, *C*_6_, *C*_7_, *C*_14_	The predicted value is obtained mainly according to the relevant planning
Category B	*C*_10_, *C*_11_, *C*_12_, *C*_13_, *C*_15_, *C*_16_	Establishing a reasonable model or adopting appropriate methods for prediction

Note: Indicators have the same meaning as in Table 1.

## 4. Results

### 4.1. Data Sources, index prediction, and parameter determination

1. Relevant parameters.
  The inflow frequencies were set to 50%, 75%, and 90%. The current year was 2018. Since the water rights trading period of Helan County is basically set for 20–25 years and is implemented from 2020, two planning years are set: 2030 (medium-term) and 2040 (long-term).
2. Sample data sources.
  Since the Chinese government proposed to implement the strictest water resource management system in 2011, to make the sample data comparable, this paper focuses on the analysis of basic data from 2012 to 2018. ① Data reflecting the natural endowment of water resources uses the average value of many years, and is mainly obtained from local water resources bulletins or water resources statistical bulletins. These dates contain total water resources, surface water, underground water, precipitation, evaporation, among other relevant factors. ② Social and economic data are obtained from governmental and departmental work reports or socio-economic statistical yearbooks, and mainly include total population, land area, GDP, industrial added value, and irrigation area. ③ Other water resources and water environmental data are mainly obtained from or are referred to relevant policy documents, planning texts, and research results of Helan County. Specific data sources mainly refer to the "Ningxia Water Resources Allocation Guarantee Plan (2016–2020)", the "Ningxia Agricultural Irrigation Water Quota" (2014), the "Ningxia Current Status of Agriculture in 2018 Report on the Results of Measurement and Analysis of the Effective Utilization Coefficient of Irrigation Water”, the “Outline of the 13th Five-Year Plan for National Economic and Social Development of Helan County", the "Thirteenth Five-Year Plan for Water Conservancy Development of Helan County" (2016), and the "Thirteenth Five-Year Plan for Agricultural Industry Development in Helan County" (2016). These are the data sources of industrial sewage discharge, domestic sewage discharge, water function area compliance rate, water supply, water consumption, effective utilization coefficient of farmland irrigation water, water consumption of 10^4^ Yuan industrial added value, and water consumption rate.
3. Prediction of relevant indicators.
  According to statistical data, the average water consumption of Helan County from 2012 to 2018 was 433.8 million m^3^, among which, 399.9 million m^3^ of water originated from the Yellow River, accounting for 92.21%; and 33.8 million m^3^ originated from groundwater, accounting for 7.79%. Among this consumption, agricultural and ecological water withdrawals reached up to 411.8 million m^3^, accounting for 94.9% of the total water consumption. Consequently, the runoff of the Yellow River trunk stream was the main water source of Helan County. Considering regional factors, the measured runoff data from 1980 to 2018 of Lanzhou hydrological station in the mainstream of the Yellow River were selected as the main basis for adjusting the change of water supply under different inflow frequencies.
  The related prediction process of category A indicators are shown in Table 3.
  Category B indicators need to build a model for prediction. Specific ideas and prediction models are listed in Table 4.
  Through the above model or method, the predict results for each indicator were obtained, as shown in Table 5.
4. The evaluation index corresponds to the evaluation standard value range.
  In reference to relevant national standards, prior literature, and in combination with the "vast land and few people" characteristics of Helan County, the evaluation index corresponding to the evaluation standard value range was determined, as shown in Table 6.
5. Determination of index weight. The combined weight is determined according to Formulas (6)–(8), as shown in Table 6.
6. Determination of Δ. Based on the analysis of existing water rights trading cases in Ningxia Autonomous Region, the allowable deviation of tradable water rights was set as 500,000 m^3^; therefore, Δ = 1 million m^3^.

**Table 3:** Forecast description of Class A indicators.

Index code	Description of prediction
*C*_1_	According to the annual average, the total amount of water resources at a 50% inflow frequency was 540.9 million m^3^, among which, 481.2 million m^3^ originated from the Yellow River and 0.597 million m^3^ originated from local water. According to the relevant plan, the total population of the planned year will be 283,200 in 2030, and 298,100 in 2040.
*C*_2_	According to the annual average, *C*_2_ is 33.90 m^3^/km^2^, among which, the total land area is 1595.5 km^2^.
*C*_3_	The annual average rainfall is 193 mm, and the evaporation is 1716.8 mm; therefore, based on the annual average, *C*_3_ is 8.895.
*C*_4_	According to the relevant planning of the government of Helan County, the urbanization rate will be about 61% in 2025. Combined with the development of the urbanization rate in the first 15 years and the annual increase rate of 1%, the urbanization rates for 2030 and 2040 are predicted to be 65% and 70%, respectively.
*C*_5_	The total population and total land area of the planning year are identical to those of as *C*_1_ and *C*_2_, respectively.
*C*_6_	The total water supply is determined in accordance with the red line of the total water intake control as stipulated in the strictest water resources management system. It is 472 million m^3^ in 2030 and 472 million m^3^ in 2004; the total population in the planning year is the same as that of *C*_1_.
*C*_7_	The total water supply and the total amount of water resources are the same as above.
*C*_8_	According to the average value, *C*_8_ is 92.21% under 50% inflow frequency.
*C*_9_	According to the average value, *C*_9_ is 7.79% under 50% inflow frequency.
*C*_14_	According to the relevant plan of the government of Helan County, *C*_14_ will be 85.0% in 2030 and 89.0% in 2040.

Note: The meaning of indicators is the same as in Table 1.

**Table 4:** Prediction description and prediction model or method of category B indicators.

Index code	Description of prediction	Prediction model or method
*C*_10_	This is divided into two steps: ① The total water consumption data was collected from 2012 to 2018 and a scatter diagram was plotted, as shown in Fig 3a. No obvious changing trend was found; therefore, model GM (1,1) is adopted for to prediction of the change of total water consumption. ② The total water consumption is used to predict the per capita water consumption.	① Prediction model of total water consumption:$\left\{\begin{array}{c} {\hat{x}}^{\left(1\right)}(k+1)=546.27e^{-0.0081k}+550.72\\ {\hat{x}}^{\left(0\right)}(k+1)={\hat{x}}^{\left(1\right)}(k+1)-{\hat{x}}^{\left(1\right)}(k) \end{array}\right.$ (*k* = 1,2,3,…,*n*) ② ${\hat{y}}_{n}={\hat{x}}^{\left(0\right)}\left(n\right)/p\left(n\right)$ Where,${\hat{y}}_{n}$ represents the predicted value of *C*_10_ phase n, ${\hat{x}}^{\left(0\right)}\left(n\right)$ represents the predicted value of water consumption, and *p*(*n*) represents the current population.
*C*_11_	The irrigation water utilization coefficients of Tanglaiqu, Xiganqu, Huinongqu, and Hanyan canal irrigation areas that flowed through Helan county from 2012 to 2018 were collected. A scatter plot of the average value of irrigation water utilization coefficient of the four irrigation areas is shown in Fig 3b, which conforms to the change rule of the modified exponential curve.	${\hat{y}}_{n}=0.613-0.230\times 0.875^{n}$ Where, ${\hat{y}}_{n}$ represents the predicted value of *C*_11_ phase n, and n represents the ordinal number of years, corresponding to 2012, n = 1.
*C*_12_	The actual value in 2018 is 13.0 m^3^ per 104 Yuan, which is far lower than the water consumption of 10^4^ Yuan of industrial added value in similar regions. According to the survey, this is not caused by technological progress, but determined by small industrial scale and low water consumption. As the sample data is not typical, the Delphi method is adopted for prediction.	Five experts, including governmental officials, university professors, and business managers, were employed. Through three rounds of questionnaire survey, statistical evaluations, and feedback, the predicted results tended to converge.
*C*_13_	The water consumption rates from 2012 to 2018 were collected and a scatter diagram was plotted, as shown in Fig 3c. This time basically belongs to a relatively stable series. Therefore, a quadratic exponential smoothing model is used to predict the trend value, and the adjustment coefficient of technological progress is introduced to correct it.	Smooth model: $\left\{\begin{array}{l} s_{t}^{\left(1\right)}=\alpha y_{t}+\left(1-\alpha \right)s_{t-1}^{\left(1\right)}\\ s_{t}^{\left(2\right)}=\alpha s_{t}^{\left(1\right)}+\left(1-\alpha \right)s_{t-1}^{\left(2\right)}\\ a_{t}=2s_{t}^{\left(1\right)}-s_{t}^{\left(2\right)}\\ b_{t}=\frac{\alpha }{1-\alpha }\left(s_{t}^{\left(1\right)}-s_{t}^{\left(2\right)}\right) \end{array}\right.$ Where, *α* represents the smooth parameter, *y*_*t*_ represents the sample value, $s_{t}^{\left(1\right)}$ and $s_{t}^{\left(2\right)}$ represent the first exponential smoothing value and the second exponential smoothing value, respectively, and $s_{0}^{\left(1\right)}$ and $s_{0}^{\left(2\right)}$ are smooth initial values. Assuming *α* = 0.25, $s_{0}^{\left(1\right)}=s_{0}^{\left(2\right)}=y_{1}$ to obtain the following prediction model:${\hat{y}}_{2018\text{+}k}=\theta_{tec}^{\left(k\right)}\left(0.453-0.0004k\right)$ Where, *k* represents the number of intervals between the planning year and 2018, and $\theta_{tec}^{\left(k\right)}$ represents the adjustment coefficient of technological progress, which will be 0.98 in 2030 and 0.94 in 2040.
*C*_15_	This is divided into two steps: ① the industrial water consumption data from 2012 to 2018 was collected and a scatter diagram was plotted, as shown in Fig 3d. This is in accordance with the changing rule of the modified exponential curve; therefore, the model was constructed to predict the change trend of industrial water consumption. ② By using the change of industrial water consumption to reflect the change of industrial wastewater discharge, and by introducing the policy adjustment coefficient to modify trend value, the industrial pollution diameter ratio can be measured.	① Prediction model of industrial water consumption: ${\hat{x}}_{n}=0.0828+0.0605\times 0.926^{n}$ Where, ${\hat{x}}_{n}$ represents the predicted value of *C*_15_ phase n, n represents the ordinal number of years, corresponding to 2012, n = 1. ② Prediction model of the industrial pollution diameter ratio: ${\hat{y}}_{n}=\theta_{pol}^{\left(n\right)}\times y_{2018}\times \frac{{\hat{x}}_{n}}{x_{2018}}$ Where, $\theta_{pol}^{\left(n\right)}$ represents the policy adjustment coefficient, which is predicted to be 0.98 in 2030 and 0.94 in 2040. *y*_2018_ was the industrial pollution diameter ratio in 2018.
*C*_16_	This is divided into two steps: ① The domestic water consumption data from 2012 to 2018 was collected and a scatter diagram was plotted, as shown in Fig 3e. This is in accordance with the changing rule of the law of linear change; therefore, the model was constructed to predict the change trend of domestic water consumption. ② By using the change of domestic water consumption to reflect the change of domestic wastewater discharge, and by introducing the policy adjustment coefficient correction trend value, the life pollution diameter ratio can be measured.	① Prediction model of domestic water consumption: ${\hat{x}}_{n}=0.075+0.0115n$ Where, ${\hat{x}}_{n}$ represents the predicted value of *C*_16_ phase n, and n represents the ordinal number of years, corresponding to 2012, n = 1. ② Prediction model of life pollution diameter ratio: ${\hat{y}}_{n}=\theta_{pol}^{\left(n\right)}\times y_{2018}\times \frac{{\hat{x}}_{n}}{x_{2018}}$ Where, $\theta_{pol}^{\left(n\right)}$ represents the policy adjustment coefficient, which is predicted to be 0.98 in 2030 and 0.94 in 2040. *y*_2018_ was the life pollution diameter ratio in 2018.

Note: Indicators have the same meaning as in Table 1.

**Fig 3:**
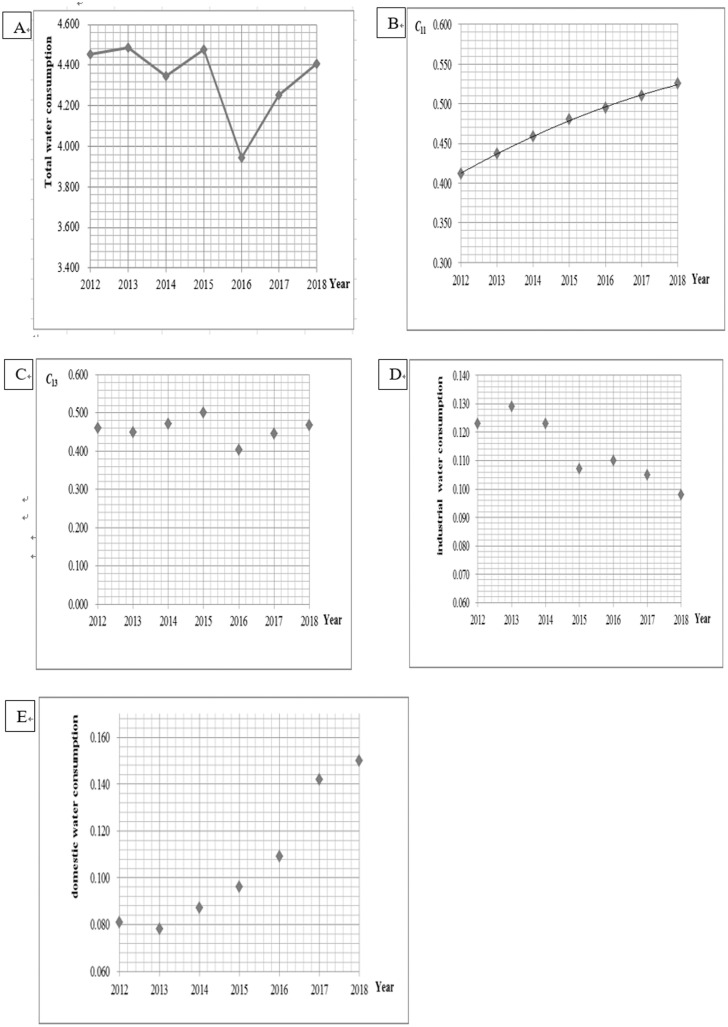
Changes of each indicator from 2012 to 2018. **(a)** Change of total water consumption. **(b)** Change of utilization coefficient of farmland irrigation water. **(c)** Change of water consumption rate. **(d)** Change of industrial water consumption. **(e)** Change of domestic water consumption.

**Table 5:** Actual value in 2018 and forecast value of indicators in 2030 and 2040 with different inflow frequencies.

Index code	Unit	Year/Water inflow frequency						
		2018	2030			2040		
			50%	75%	90%	50%	75%	90%
*C*_1_	m^3^/person	2066.87	1909.95	1680.75	1470.66	1814.49	1596.75	1397.15
*C*_2_	m^3^/km^2^	33.90	33.90	33.90	33.90	33.90	33.90	33.90
*C*_3_	/	8.90	8.90	8.90	8.90	8.90	8.90	8.90
*C*_4_	%	56.98	65.00	65.00	65.00	70.00	70.00	70.00
*C*_5_	Person/km^2^	164.02	177.50	177.50	177.50	186.84	186.84	186.84
*C*_6_	m^3^/ person	1792.13	1666.67	1466.67	1283.34	1583.36	1393.35	1219.19
*C*_7_	%	86.70	87.26	76.78	66.42	87.26	76.78	66.42
*C*_8_	%	92.21	92.21	91.23	90.11	92.21	91.23	90.11
*C*_9_	%	7.79	7.79	8.77	9.89	7.79	8.77	9.89
*C*_10_	m^3^/ person	1684.37	1556.77	1556.77	1556.77	1478.93	1478.93	1478.93
*C*_11_	/	0.53	0.59	0.59	0.59	0.60	0.60	0.60
*C*_12_	m^3^/10^4^CNY	13.0	30.0	30.0	30.0	35.0	35.0	35.0
*C*_13_	%	46.73	43.92	43.92	43.92	42.13	42.13	42.13
*C*_14_	%	78.6	85.02	85.02	85.02	88.01	88.01	88.01
*C*_15_	%	0.71	0.71	0.71	0.71	0.56	0.56	0.56
*C*_16_	%	2.72	2.51	2.51	2.51	2.42	2.42	2.42

Note: Indicators have the same meaning as in Table 1.

**Table 6:** Index weights and corresponding evaluation standard value ranges.

index code	Gradation					Weight
	Lower	Low	Medium	High	Higher	
*C*_1_	>3000	2000	1000	500	<500	0.081
*C*_2_	>50	40	30	20	<20	0.080
*C*_3_	<1	1.25	2.5	4	>4	0.079
*C*_4_	<30	45	60	75	>75	0.039
*C*_5_	<10	50	150	250	>250	0.041
*C*_6_	>1500	1000	500	200	<200	0.084
*C*_7_	>85	70	50	25	<25	0.083
*C*_8_	>95	90	85	80	<80	0.019
*C*_9_	>5	3.5	2	1	<1	0.018
*C*_10_	<300	400	500	600	>600	0.095
*C*_11_	>0.65	0.6	0.55	0.5	<0.5	0.066
*C*_12_	<20	40	60	80	>100	0.068
*C*_13_	<20	30	40	50	>50	0.065
*C*_14_	>95	80	70	60	<60	0.062
*C*_15_	<10	20	25	30	>30	0.061
*C*_16_	<5	7	10	15	>15	0.059

Note: The meaning of indicators is the same as in Table 1. Among them, C1 was classified according to the "United Nations Standard for Measuring National Wealth", C2 and C11 were classified according to "Water Resources Evaluation Guidelines SL/T238-199" and were adjusted according to the Characteristics of Ningxia; C3 was graded according to the "Natural Zoning Standard of Chinese Academy of Sciences"; C4 and C14 were graded according to “the National Civilized City Evaluation Manual;” C5 was graded according to “China’s Urban Hierarchy Standard”; C6 was classified according to reference [26] and was adjusted according to the characteristics of Ningxia; the grade classifications of C7, C8, C9, C13, C15, C16 were classified according to reference [27], and C10 and C12 were graded in reference to “Guidelines for Water Index Evaluation GB-T7119-1993”.

### 4.2. Analyzing the risk evolution trend of the shortage of water resources in Helan County without water rights trading

**Fig 4:**
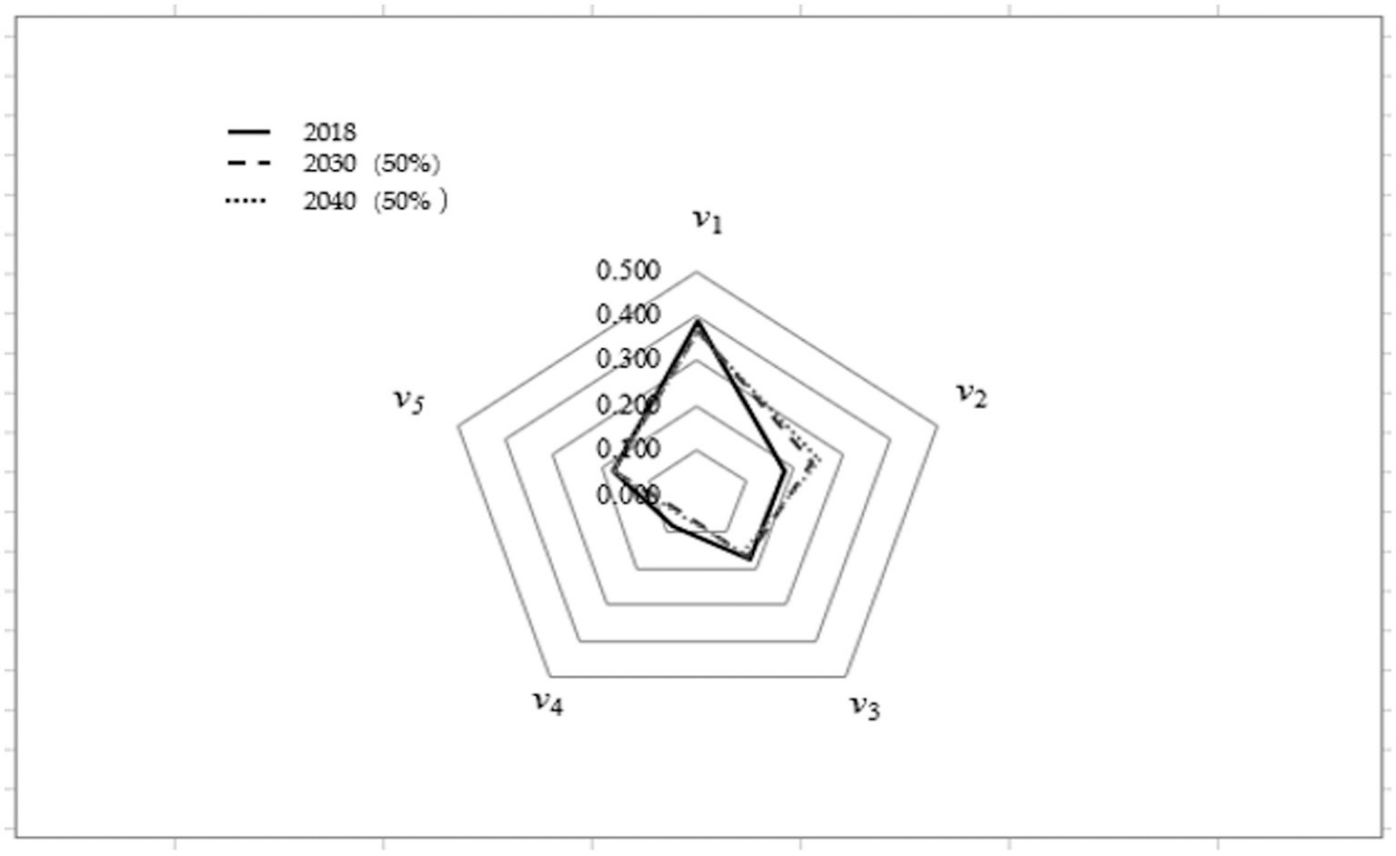
Comparison of evaluation grades for three years without water right trading.

1. Water shortage risk in 2018
  According to the data in Table 2, the grading interval shown in Table 3, and Formulas (3)–(5), *r*_*ij*_ can be obtained as follows:
  $$R^{\text{T}}=\left[\begin{array}{cccccccccccccccc} 0.066 & 0 & 0 & 0 & 0 & 1 & 1 & 0.558 & 1 & 0 & 0 & 1 & 0 & 0 & 1 & 1\\ 0.934 & 0.390 & 0 & 0.151 & 0 & 0 & 0 & 0.442 & 0 & 0 & 0 & 0 & 0 & 0.930 & 0 & 0\\ 0 & 0.610 & 0 & 0.849 & 0.860 & 0 & 0 & 0 & 0 & 0 & 0.500 & 0 & 0.327 & 0.070 & 0 & 0\\ 0 & 0 & 0 & 0 & 0.140 & 0 & 0 & 0 & 0 & 0 & 0.500 & 0 & 0.673 & 0 & 0 & 0\\ 0 & 0 & 1 & 0 & 0 & 0 & 0 & 0 & 0 & 1 & 0 & 0 & 0 & 0 & 0 & 0 \end{array}\right]$$
  According to the weight of Table 3, and Formula (2), *B* = (0.389,0.179,0.176,0.082,0.174). This means that the evaluation grade is *v*_1_.
2. Water shortage risk without water rights trading in 2030
  Similarly, the water shortage risk assessment grade in 2030 without water right trading would be as follows: Under a water inflow frequency of 50%, *B* = (0.370, 0.237, 0.168, 0.050, 0.174), the evaluation grade is *v*_1_; under a water inflow frequency of 75%, *B* = (0.317, 0.273, 0.185, 0.051, 0.174), the evaluation level is *v*_1_; under a water inflow frequency of 90%, *B* = (0.253, 0.337, 0.185, 0.051, 0.174), the evaluation level is *v*_2_.
3. Water shortage risk without water rights trading in 2040
  Similarly, the water shortage risk assessment grade in 2040 without water right trading would be as follows: Under a water inflow frequency of 50%, *B* = (0.366, 0.251, 0.154, 0.055, 0.174), the evaluation grade is *v*_1_; under a water inflow frequency of 75%, *B* = (0.301, 0.269, 0.201, 0.055, 0.174), the evaluation level is *v*_1_; under a water inflow frequency of 90%, *B* = (0.239, 0.331, 0.201, 0.055, 0.174), the evaluation level is *v*_2_.
4. Evolution trend analysis
  The evaluation result for 2018 is compared with the predicted results for 2030 and 2040 under the water inflow frequency of 50%, and a radar chart is drawn, as shown in Fig 4. Fig 4 shows that the risk grade of water resource shortages in 2018, 2030, and 2040 all maintained the *v*_1_ level, but a trend of "gravity shift backward" exists among the five evaluation levels. This means that the value of *v*_1_ tends to decrease, while the value of *v*_2_ tends to increase.

### 4.3. Calculation of tradable water rights in 2030 based on risk analysis of water shortage

Based on the impact water rights trading exerts on the risk of water shortage in Helan County in 2030, using MATLAB (version 7.01), the simulated annealing algorithm and the theoretical values of the amount of tradable water rights under the water inflow frequency of 50%, 75%, and 90% were calculated. The process of increasing and decreasing is calculated by equivalent method, and Δ = 1 million m^3^.

1. Under a water inflow frequency of 50%, the corresponding index value is obtained by Formulas (11) and (12), and then based on the process shown in Fig 1, the amount of tradable water rights is calculated. Finally, after iteration, based on Formula (14), the theoretical value of the amount of tradable water rights was obtained as 75 million m^3^.
2. Similarly, under a water inflow frequency of 75%, after iteration, the theoretical value of the amount of tradable water rights is 15 million m^3^.
3. Under a water inflow frequency of 90%, since the water resource supply quantity has exceeded the bottom line of water demand, Helan County cannot provide the required amount of tradable water rights anymore. This means that Helan County, as the transferor, has a theoretical value of the amount of tradable water rights of 0.

### 4.4. Calculation of the amount of tradable water rights in 2040 based on risk analysis of water shortage

Similarly, it can be calculated that:

1. Under a water inflow frequency of 50%, the corresponding index value can be obtained by Formulas (11) and (12). Then, based on the process shown in Fig 1, the amount of tradable water rights can be calculated. Finally, after iteration, based on Formula (14), the theoretical value of the amount of tradable water rights is obtained as 65 million m^3^.
2. Under a water inflow frequency of 75%, after iteration, the theoretical value of the amount of tradable water rights is 17 million m^3^.
3. Under a water inflow frequency of 90%, similarly, Helan County also cannot provide the required amount of tradable water rights. This means that Helan County, the transferor, has a theoretical value of the amount of tradable water rights of 0.

**Fig 5:**
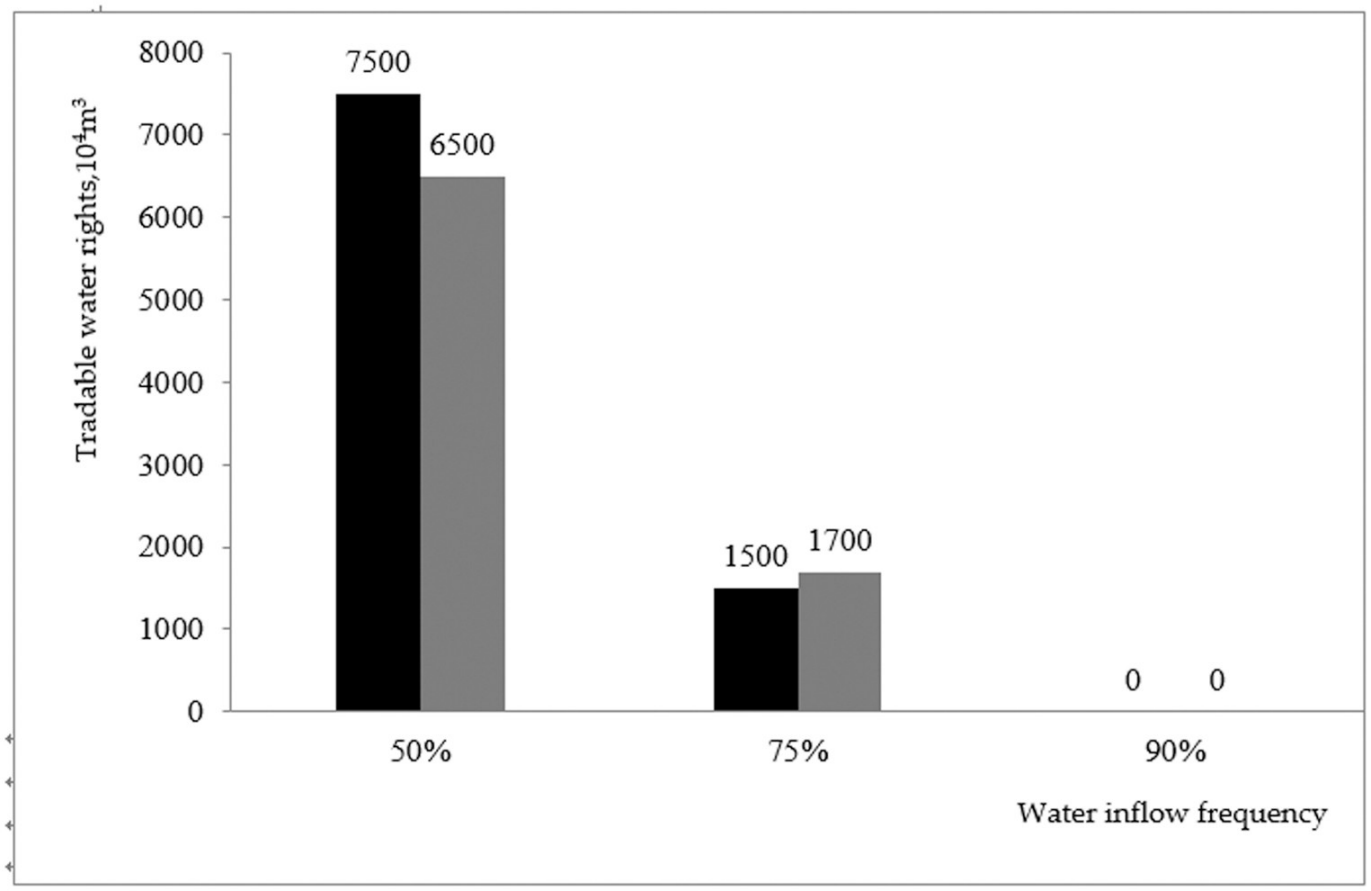
Comparison of theoretical values of the amounts of tradable water rights in different planning years and scenarios.

The theoretical values of the amount of tradable water rights under three scenarios in the two planning years are compared and analyzed, as shown in Fig 5.

### 4.5 Determination of the recommended value of tradable water rights

According to the above analysis, the following conclusions can be presented. During the implementation of water right trade in Helan County, to maintain a "low" level of risk in 2030, the theoretical values of tradable water rights are 75 million m^3^ and 15 million m^3^, under 50% and 75% water frequencies, respectively. There no tradable water rights exist under a 90% frequency. Similarly, to ensure that the risk classification in 2040 maintains "low" or "lower" levels, the theoretical value is 75 million m^3^ under 50% frequency and 17 million m^3^ under 65% frequency. Tradable water shortage exists at a frequency of 95%.

1. Helan County needs to control the water shortage crisis that is simultaneously caused by water right trading in 2030 and 2040. Therefore, the intersection of the calculation results of the two planning years should be implements with relatively strict standards.
2. The theoretical values of tradable water rights in Helan County vary greatly under different water frequencies. It is therefore necessary to not only consider the adverse effects induced by natural factors, but also the driving factors of completed transactions to both society and economy. Therefore, given the existence of episodes of extreme situations under 90% frequency, it is more reasonable and realistic to apply calculation results with 50% and 75% water frequencies as the main basis to determine the amount of tradable water rights.

In summary, the amount of tradable water rights in Helan County should be controlled at [15 million m^**3**^, 65 million m^**3**^], and the recommended value is 40 million m^**3**^ using the mean value method.

## 5. Discussion

### 5.1 Comparison with other weight calculation methods

The subjective and objective combination method was adopted to obtain the weight of water shortage risk assessment indicators; then, the tradable water rights were calculated. To test the possible effects of differently weighted calculation methods on the tradable water rights measurement results, the subjective weight measurement methods CRITIC and CRITIC-M, as well as the objective weight measurement methods LBWA and FUCOM [28–30] were used to calculate the weights. Then, the corresponding tradable water rights were calculated, and the changes in the results of tradable water rights were analyzed.

#### 5.1.1 Four other types of weight calculation methods

1. The CRITIC method. To improve the accuracy of the calculation, the evaluation index values of five different counties located in the upper reaches of the Yellow River were collected. Helan County was used as the case study, and six schemes were established for analysis and calculation. The following steps were taken: ① Constructing the initial decision matrix *X* = [*ξ*_*ij*_]_*m*×*n*_, where *ξ*_*ij*_ represents the attribute value of index *j* of scheme *i*; ② Normalizing the index value. To maximize criteria, $\xi_{ij}=\frac{\xi_{ij}-\xi_{j}^{\text{min}}}{\xi_{j}^{\text{max}}-\xi_{j}^{\text{min}}},\;i=1,2,\ldots ,n;\;j=1,2,\ldots ,m$ was used; to minimize criteria, $\xi_{ij}=\frac{\xi_{j}^{\text{max}}-\xi_{ij}}{\xi_{j}^{\text{max}}-\xi_{j}^{\text{min}}},\;i=1,2,\ldots ,n;\;j=1,2,\ldots ,m;$ where $\xi_{j}^{\text{max}}=\underset{j}{\text{max}}\left\{\xi_{1j},\xi_{2j},\ldots ,\xi_{mj}\right\};\xi_{j}^{\text{min}}=\underset{j}{\text{min}}\left\{\xi_{1j},\xi_{2j},\ldots ,\xi_{mj}\right\}$ was used; ③ Calculating the standard deviation *σ*_*j*_; ④ Constructing the matrix *L* = [*l*_*jk*_]_*n*×*n*_, containing the coefficients of linear correlation of vectors *ξ*_*j*_ and *ξ*_*k*_; ⑤ $w_{j}=\frac{W_{j}}{\sum_{k=1}^{m}W_{k}}$ is used to calculate the weights, where $\varphi_{j}=\sum_{k=1}^{n}\left(1-l_{jk}\right)$ and *W*_*j*_ = *σ*_*j*_⋅*φ*_*j*_.
2. The CRITIC-M method. The data of the same six schemes are also used for analysis and calculation in this case. The following steps are taken: ① Constructing the initial decision matrix *X* = [*ξ*_*ij*_]_*m*×*n*_, where *ξ*_*ij*_ represents the attribute value of index *j* of scheme *i*; ② To normalize the index value, and maximize criteria, $\xi_{ij}=\frac{\xi_{ij}}{\xi_{j}^{\text{max}}},\;i=1,2,\ldots ,n;\;j=1,2,\ldots ,m;$ where $\xi_{j}^{\text{max}}=\underset{j}{\text{max}}\left\{\xi_{1j},\xi_{2j},\ldots ,\xi_{mj}\right\}$ is used; to minimize criteria, $\xi_{ij}=-\xi_{ij}^{*}\text{+}\xi_{ij}^{*\text{max}}\text{+}\xi_{ij}^{*\text{min}};\;i=1,2,\ldots ,n;\;j=1,2,\ldots ,m;$ where $\xi_{j}^{*\text{max}}=\underset{j}{\text{max}}\left\{\xi_{1j}^{*},\xi_{2j}^{*},\ldots ,\xi_{mj}^{*}\right\};\;\xi_{j}^{*\text{min}}=\underset{j}{\text{min}}\left\{\xi_{1j}^{*},\xi_{2j}^{*},\ldots ,\xi_{mj}^{*}\right\};$ is used. ③ Calculating the standard deviation *σ*_*j*_; ④Formula $w_{j}=\frac{\frac{\bar{\xi_{j}}}{1-\xi_{j}}\cdot \sigma_{j}}{\sum_{j=1}^{n}\left(\frac{\bar{\xi_{j}}}{1-\xi_{j}}\cdot \sigma_{j}\right)}$ is used to calculate the weights, where $\bar{\xi_{j}}=\frac{1}{m}\sum_{i=1}^{n}\xi_{ij}$.
3. The LBWA method. Ten experts were invited to form a panel of experts. These experts mainly came from the China Water Rights Exchange, local water resources trading center, university professors, and the water administration department. The following steps were taken: ① The most important criterion was determined from the set of criteria *S* = {*C*_1_, *C*_2_, …, *C*_16_}. In the defined problem, criterion *C*_10_ was selected as the most important/influential criterion; ② criteria are grouped by their levels of significance. In accordance with the preferences of decision makers, the criteria are grouped in the following subsets/levels: *S*_1_ = {*C*_10_, *C*_1_, *C*_2_, *C*_3_, *C*_6_, *C*_7_}, *S*_2_ = {*C*_11_, *C*_12_, *C*_13_, *C*_14_, *C*_15_, *C*_16_}, *S*_3_ = {*C*_4_, *C*_5_}, and *S*_3_ = {*C*_8_, *C*_9_}; ③ The maximum value of the scale is defined for comparing the criteria; let *r* = 6. Based on the preferences of decision makers, the following relationships can be defined: Level *S*_1_, *I*_10_ = 0, *I*_6_ = *I*_7_ = 1, *I*_1_ = *I*_2_ = 2, *I*_6_ = 3; Level *S*_2_, *I*_11_ = *I*_12_ = *I*_13_ = *I*_14_ = *I*_15_ = 1, *I*_16_ = 2; Level *S*_3_, *I*_4_ = *I*_5_ = 1; Level *S*_4_, *I*_8_ = *I*_9_ = 4. ④ Let *r*_0_ = 9 and calculate the corresponding value *f*(*C*_*i*_); ⑤ the weight *w*_*i*_ is calculated.
4. The FUCOM method. The 10 experts mentioned above were also employed for this method. The following steps were taken: ① The decision-makers ranked the criteria as: *C*_10_ > *C*_3_ > *C*_2_ > *C*_1_ > *C*_7_ > *C*_6_ > *C*_11_ > *C*_12_ > *C*_13_ > *C*_14_ > *C*_15_ > *C*_16_ > *C*_4_ > *C*_5_ > *C*_8_ > *C*_9_; ② Based on the decision-makers’ preferences, the comparative priorities of the ranked criteria were determined and the vector of the comparative priorities of the evaluation criteria was obtained as listed in Table 7.

**Table 7:** Vector of the comparative priorities of the evaluation criteria.

Criteria	*C*_10_	*C*_3_	*C*_2_	*C*_1_	*C*_7_	*C*_6_	*C*_11_	*C*_12_
*φ*_*k*⁄(*k*+1)_	1.000	1.111	1.059	1.012	1.012	1.012	1.171	1.077
Criteria	*C*_13_	*C*_14_	*C*_15_	*C*_16_	*C*_4_	*C*_5_	*C*_8_	*C*_9_
*φ*_*k*⁄(*k*+1)_	1.083	1.034	1.055	1.100	1.111	1.125	1.143	1.167

③ According to $\frac{w_{k}}{w_{k+1}}=\varphi_{k/\left(k+1\right)}$ and $\frac{w_{k}}{w_{k+2}}=\varphi_{k/\left(k+1\right)}⊗\varphi_{\left(k+1\right)/\left(k+2\right)}$, the relationship between index weights should be calculated; ④ the optimization model was constructed and solved to obtain the index weight

$$\begin{array}{l} \text{min}\;\chi \\ s.t.\\ \left|\frac{w_{j\left(k\right)}}{w_{j\left(k+1\right)}}-\varphi_{k/\left(k+1\right)}\right|\leq \chi ,\forall j\\ \left|\frac{w_{j\left(k\right)}}{w_{j\left(k+2\right)}}-\varphi_{k/\left(k+1\right)}⊗\varphi_{\left(k+1\right)/\left(k+2\right)}\right|\leq \chi ,\forall j\\ \sum_{j=1}^{n}w_{j}=1,\forall j\\ w_{j}\geq 0,\forall j \end{array}$$

The weights calculated by the four methods are shown in Table 8:

**Table 8:** Weights calculated by the four methods.

Index code	Method / Weight			
	CRITIC	CRITIC-M	LBWA	FUCOM
*C*_1_	0.085	0.087	0.090	0.081
*C*_2_	0.093	0.095	0.090	0.082
*C*_3_	0.058	0.055	0.082	0.087
*C*_4_	0.050	0.052	0.035	0.044
*C*_5_	0.046	0.043	0.035	0.039
*C*_6_	0.084	0.087	0.099	0.079
*C*_7_	0.096	0.098	0.099	0.080
*C*_8_	0.024	0.022	0.025	0.034
*C*_9_	0.028	0.024	0.025	0.029
*C*_10_	0.075	0.075	0.111	0.097
*C*_11_	0.064	0.067	0.052	0.068
*C*_12_	0.070	0.073	0.052	0.063
*C*_13_	0.053	0.052	0.052	0.058
*C*_14_	0.051	0.048	0.052	0.056
*C*_15_	0.062	0.060	0.052	0.053
*C*_16_	0.061	0.062	0.049	0.048

#### 5.1.2 Comparative analysis of water rights trading volumes under different weights

In accordance with the inflow frequency, 50%, 75%, and 90% of water rights were assumed, and the weights were calculated (as shown in Table 8) and were used according to the process shown in Fig 1. The theoretical value of tradable water rights in 2030 and 2040 was calculated by using Formula (14), as shown in Table 9. For the convenience of comparison, the theoretical value of water right trading volume, calculated by combination weight above, is listed in Table 9.

**Table 9:** Theoretical values of water right trading volume under four weights (10^4^ m^3^).

Method	Year/Water inflow frequency					
	2030			2040		
	50%	75%	90%	50%	75%	90%
CRITIC	7800	1700	0	6500	1400	0
CRITIC-M	7400	1500	0	5500	1300	0
LBWA	7000	1500	0	6500	1500	0
FUCOM	7000	1500	0	5500	1000	0
Combination method	7500	1500	0	6500	1700	0

1. The calculation results of 2030 show that: ① At 50% incoming water frequency, the result calculated by the CRITIC method is 78 million m^3^, which implies an increase of 3 million m^3^ over the original result, representing an increase of 4%. The results obtained by the other three methods are all lower than the original results, where the LBWA and FUCOM methods are both reduced by 5 million m^3^, representing a reduction ratio of 6.7%, and the CRITIC-M method is reduced by 1 million m^3^, representing a reduction ratio of less than 2%. ② At 75% water inflow frequency, the result calculated by the CRITIC method is 17 million m^3^, which is 2 million m^3^ more than the original result, representing an increase of 13.3%. The results obtained by the other three methods are not different compared with the original results. ③ Under a water inflow frequency of 90%, the calculation results do not change.
2. The calculation results of 2040 show that: ① The results calculated by CRITIC and LBWA at 50% incoming water frequency did not change compared with the original results. The results calculated by CRITIC-M and FUCOM are both 55 million m^3^, implying a decrease by 10 million m^3^, with a reduction ratio of 15.4%. ② In case of an incoming water frequency of 75%, the results calculated by the LBWA method did not change compared with the original results. The results measured by CRITIC, CRITIC-M, and FUCOM methods are reduced by 1 million m^3^, 2 million m^3^, and 5 million m^3^, respectively, compared with the original results. The associated reduction rates are 6.7%, 13.3%, and 33.3%, respectively. ③ Under a water inflow frequency of 90%, the calculation results did not change.

In general, the theoretical value of the water right trading volume calculated by these four weight calculation methods does not significantly deviate from the original results except in specific cases. This shows that the model proposed in this paper has strong robustness. To control the measurement error, this paper suggests that in addition to the FUCOM method, the other three weight calculation methods CRITIC, CRITIC-M, and LBWA can also be selectively used in practical applications.

### 5.2 Comparative analysis of tradable water rights based on the water-saving potential

According to the “Water Rights Trade Indicator Analysis Report in Helan Country of China”, the amount of water-saving in the status year and the water-saving potential in the planning year of Helan County can be calculated as follows:

1. In the current year, the annual average water-saving amount from 2012 to 2019 was 13.6839 million m^3^, where 9.45 million m^3^ originate from governmental investment in slab-lined canals and efficient water-saving irrigation projects; 423 million m^3^ originate from water users’ investment in water-saving irrigation projects.
2. For the planning year of 2045, the comprehensive water-saving potential is 95.46 million m^3^ based on the right index. The approach of subentry calculating the total potential is 63.6302 million m^3^, 56.6187 million m^3^ of which originates from the government’s water-saving reform engineering on irrigated areas, and 7011500 m^3^ originates from water users’ efficient water-saving irrigation projects. In Helan County, the water-saving potential mostly originates from agriculture, and the key measurement index data include the reduction of rice planting areas and an increased efficiency of water-saving in irrigated areas. The rice planting area in Helan County for the planning year will be controlled within 129500 mu, which represents a decrease by 57500 mu compared with the current situation; the proportion of the efficient water-saving irrigation area will increase by 40%, i.e., to 235800 mu.

From the perspective of controlling the water shortage risk of transferors, the amount of tradable water rights of Helan County is [15 million m^3^, 365 million m^3^]. The upper end of this interval is very close to the water-saving potential of 63.6302 million m^3^, as calculated by sub items; however, it differs from the value 95.46 million m^3^, which was obtained based on the right index. The main reason is that under the right index allocation, the right confirmation quantity holds a margin of the actual water supply in the current year. Only part of the water-saving potential can be used for water rights transactions; consequently, the suggested value of the tradable water rights is 40 million m^3^, retaining approximately 2/3 of the water saving potential of 63.6302 million m^3^. This proportion is in line with mainstream beliefs, and further verifies the feasibility to control transferors’ risk of water shortage. These results show that the method proposed in this paper forms a good complementary and corroborative relationship with that of using the water-saving potential of the transferor to calculate the amount of tradable water rights.

## 6. Conclusion

From the perspective of controlling the transferors’ risk of water shortage, this paper calculates the amount of tradable water rights and discusses the settings under different water frequencies. Helan County of Ningxia Autonomous Region, China, is taken as example, and the results are summarized in the following: (1) Under the scenario of no water rights trading, the risk of water shortage in Helan County is low and is affected by different water frequencies. At a frequency of 90%, the risk will be lower. (2) The amount of tradable water rights, which can be obtained by setting transferor’s risk threshold of water resources shortage, can effectively guarantee the transferor’s own socio-economic development and the security of ecological water use. Moreover, this amount can avoid the water shortage risk, water right disputes caused by excessive water trade, and can effectively promote the stability and durability of the water property right transaction market.

Suggestions are proposed in accordance with the study results. First, scientific calculation of the amount of tradable water rights is key to ensure the smooth implementation of regional water rights transactions. It is therefore necessary to consider the natural and social carrying capacities of both parties to identify the amount of tradable water rights. Of particular importance are the transferor’s socio-economic development and the security of ecological water use. The most reasonable amount of tradable water rights can be calculated by setting the water shortage risk threshold of the transferor. Moreover, the water rights trading period is longer than the current water rights trading cases in China. The risk threshold of water resources shortage can be set as either medium-term or long-term according to the actual situation of the transferor. To promote the future development of both parties of the transaction, it is significant to reduce the water shortage risk associated with water rights trading, and to ensure the promotion of regional social and economic development.

The method proposed in this paper faces certain limitations, mainly with regard to the following two points: First, the accuracy of the prediction of the mid-term and long-term evaluation indexes affects whether the measurement results of the trading volume of water rights are reasonable. The solution of this issue is to collect a sufficient number of samples and to choose reasonable prediction methods, thus improving the accuracy of the prediction. Second, the theoretical value of the water right trading volume is affected by different incoming water frequencies. To determine the recommended value of water right trading volume, the average of the measured results of two inflow frequencies (50% and 75%) should be assumed as the recommended value of tradable water rights. Whether this proposed method is applicable to different regions remains to be tested in practice.

The method proposed in this paper provides a new way to reasonably determine the scale of water rights traded by both parties. This method can compensate for the defects of the existing methods and provide both a theoretical basis and decision-making reference for the scientific measurement of tradable water rights when both parties sign a water rights transaction agreement.

## Supporting information

S1 Data (DOCX)
