# Supplementary material for: Calculation model for the amount of tradable water rights based on water shortage risk evaluation
Source: PLoS One. 2021 Aug 25;16(8):e0254428. doi: 10.1371/journal.pone.0254428 (PMC8386845; doi:10.1371/journal.pone.0254428)
Supplement: S1 Data — (DOCX) [file pone.0254428.s001.docx]

**Table 5.** Actual value in 2018 and forecast value of indicators in 2030 and 2040 with different inflow frequencies

| **Index code** | **Unit** | **Year/Water inflow frequency** | | | | | | |
| --- | --- | --- | --- | --- | --- | --- | --- | --- |
|  |  | 2018 | 2030 | | | 2040 | | |
|  |  |  | 50% | 75% | 90% | 50% | 75% | 90% |
| *C*_1_ | m^3^/person | 2066.87 | 1909.95 | 1680.75 | 1470.66 | 1814.49 | 1596.75 | 1397.15 |
| *C*_2_ | m^3^/km^2^ | 33.90 | 33.90 | 33.90 | 33.90 | 33.90 | 33.90 | 33.90 |
| *C*_3_ | / | 8.90 | 8.90 | 8.90 | 8.90 | 8.90 | 8.90 | 8.90 |
| *C*_4_ | % | 56.98 | 65.00 | 65.00 | 65.00 | 70.00 | 70.00 | 70.00 |
| *C*_5_ | Person/km^2^ | 164.02 | 177.50 | 177.50 | 177.50 | 186.84 | 186.84 | 186.84 |
| *C*_6_ | m^3^/ person | 1792.13 | 1666.67 | 1466.67 | 1283.34 | 1583.36 | 1393.35 | 1219.19 |
| *C*_7_ | % | 86.70 | 87.26 | 76.78 | 66.42 | 87.26 | 76.78 | 66.42 |
| *C*_8_ | % | 92.21 | 92.21 | 91.23 | 90.11 | 92.21 | 91.23 | 90.11 |
| *C*_9_ | % | 7.79 | 7.79 | 8.77 | 9.89 | 7.79 | 8.77 | 9.89 |
| *C*_10_ | m^3^/ person | 1684.37 | 1556.77 | 1556.77 | 1556.77 | 1478.93 | 1478.93 | 1478.93 |
| *C*_11_ | / | 0.53 | 0.59 | 0.59 | 0.59 | 0.60 | 0.60 | 0.60 |
| *C*_12_ | m^3^/10^4^CNY | 13.0 | 30.0 | 30.0 | 30.0 | 35.0 | 35.0 | 35.0 |
| *C*_13_ | % | 46.73 | 43.92 | 43.92 | 43.92 | 42.13 | 42.13 | 42.13 |
| *C*_14_ | % | 78.6 | 85.02 | 85.02 | 85.02 | 88.01 | 88.01 | 88.01 |
| *C*_15_ | % | 0.71 | 0.71 | 0.71 | 0.71 | 0.56 | 0.56 | 0.56 |
| *C*_16_ | % | 2.72 | 2.51 | 2.51 | 2.51 | 2.42 | 2.42 | 2.42 |
